# Survival of massive mesenteric infarction through midgut resection and duodenocolostomy—a case report

**DOI:** 10.1007/s00384-015-2186-x

**Published:** 2015-04-01

**Authors:** Zhan-Fei She, Xiu-Feng Yang, Lu Ma, Bo Wu, Jun-Bing Zhang, Huai-Ming Wang, Yi Lv

**Affiliations:** 1 Department of General Surgery, Ordos Central Hospital, Ordos, 017000 Inner Mongolia China; 2 Department of Hepatobiliary Surgery, The First Affiliated Hospital of Medical College, Xi’an Jiaotong University, Xi’an, 710061 Shaanxi China

Dear Editor:

Short bowel syndrome (SBS) is a common consequence of small-bowel resection that is associated with severe malabsorption, malnutrition, imbalance of water electrolytes, and metabolism disorder. Patients with SBS always require long-term parenteral nutrition to support the transition and re-adaption of intestinal absorption for nutritional autonomy. The prognosis of SBS also depends on the anatomical identity of the residual small intestine and the length of the residual colon. Salvage of the ileocecal valve is also considered critical whereas individual differences exist. In cases when small-bowel resection is inevitable, the selection of the surgical procedure and management of post-operative nutrition appear to be of cardinal importance. However, factors that influence outcome and can guide surgical and post-operative management remain obscure because of the limited number of cases presented in literature.

A 37-year-old man with abdominal angina 20 h after dinner was admitted to our department. He had abdominal distention and naupathia. The patient had a normal medical history, and there was no notable family history of relevant diseases. Physical examination showed abdominal bloating and tenderness, rebound tenderness, passive muscle tension, and an absence of bowel sounds. Enhanced abdominal computed tomography (CT) suggested superior mesenteric artery root embolism, enteric necrosis, and massive ascites. Other laboratory results were the following: white blood cell count (WBC) 30,000/μL, buffer excess −10, and an albumin of 27.7 g/L. While fluid resuscitation commenced, emergency laparotomy was performed under anesthesia and massive bloody ascites were observed. The intestine from the Treitz ligament to the transverse colon (jejunum, ileum, ileocecum, ascending colon, and the right of the transverse colon) presented in black. Gangrene had set in and became putrid with foul odor. No pulsation was observed with respect to the superior mesenteric artery root. The patient was diagnosed with embolism of the superior mesenteric artery, provoking extensive intestinal necrosis.

Given that the disease onset was more than 24 h ago, the obvious systemic endotoxicity and the instability of vital signs, it was decided to perform the operation without prior angiogramic investigation. The necrotic bowel and mesentery were excised, proximal to the caesurae in the jejunum at the Treitz ligament and distal to the middle of the left transverse colon. End-to-side anastomosis of the duodenojejunal flexure and the left half of the transverse colon was carried out using a tube-type stapler. The cut at the transverse colon was then folded and bilaterally sutured. The patient was subsequently transferred to the intensive care unit, and nutritional support was administered through a subclavian vein catheter at 24 kcal/kg, mainly of Kabiven™ PI. On day 4 post-operation, the patient started defecating and the bowel was emptied of pre-operational material. Starting on day 14, the patient began taking fluid foods and gradually switched to normal solid food intake. The patient developed early complications including hypoproteinemia, hypokalemia, diarrhea, deep vein infection, and cholestatic hyper bile academia. The patient experienced Wernicke’s encephalopathy at 6 months post-operation manifested as different degrees of coma and was relieved by intravenous vitamin B_1_ supplementation. The patient is now (~10 months post-operation) autonomous with regard to both enteral and parenteral nutrition. He maintains a healthy weight and is in good mental state, awaiting small-bowel transplantation.

The disease progression of acute superior mesenteric artery embolism proceeds rapidly, and immediate bowel resection is usually required which will result in SBS. The intestinal absorption area will be greatly reduced, and the transit time in intestine will be shorter, leading to serious malnutrition and disturbing uptake of water, electrolytes, and trace elements. The SBS-associated mortality is high, particularly after total small-bowel resection, and survival depends largely on appropriate post-operative parenteral nutrition support. In addition, strategies chosen for digestive tract reconstruction and manipulation to prevent complications are also critical to patient survival. Unfortunately, no clear guidelines, especially with regard to surgery, have been defined although successful digestive tract reconstruction through duodenal colostomy, stomach transverse anastomosis, and duodenal transverse anastomosis has been described. The present case offers a further contribution in this respect.

Important to note in the context of the current case is that the initial emergency operation revealed necrosis of the entire small intestine and the right colon of the patient. Given this life-threatening condition, resection of the midgut, including the whole small bowel and right colon, was decided upon. The team then was confronted with two surgical options, to perform duodenal colostomy or to perform digestive tract reconstruction. The first is to close the end of the residual transverse colon but needs a second reconstructive operation. We thought that closing the residual end of the duodenum might result in the formation of an artificial blind loop. Jejunum resection at the Treitz ligament of the duodenum would also make it difficult to exteriorize sufficient duodenum outside the abdominal wall for fistulation. In addition, as energy scavenging and fluid absorption critically depend on absorption in the colon, duodenum fistulation may cause severe loss of digestive juice, resulting in unmanageable water and electrolyte problems. The possible reflux of bile and other pancreatic secretions into the stomach and subsequent re-entry into the colon may also bring obvious post-operative discomfort. Hence, we favored immediate digestive tract reconstruction. Our success in dealing with the pathology at hand suggests that in future cases this may be an appropriate strategy.

In order to prevent anastomotic fistula, we first resected the Treitz ligament to mobilize sufficient duodenum and to ensure that the anastomosis would not be subjected to significant tension. The end-to-side anastomosis of the duodenum and transverse colon would also warrant good blood circulation. The application of a tube-shaped anastomat and intermittent full-thickness stitching with absorbable suturing further reinforced the anastomosis. These operations ensured no anastomotic leakage during the post-operative course and guaranteed the patient’s safety. The patient began eating food at 2 weeks post-surgery with no nausea and vomiting reported, suggesting no regurgitation of bile and pancreatic juice. Digestive tract imaging showed normal gastric emptying. The patient started post-operative defecation (five to eight times per day) and feeding fast. The diarrhea could be tolerated and did not require the use of antidiarrhea medication. Electrolyte levels were controllable through regular electrolyte supplementation while parental protein supplementation was also necessary. No severe weight loss was observed.

In conclusion, our case showed that duodenal transverse anastomosis may be considered for some patients. This surgery is associated with relatively easy post-operative management and can serve as a bridge to small-bowel transplantation. With parental nutrition support, our patient maintained a good quality of life so far up to 10 months. A long-term follow-up is needed to evaluate the outcome of this surgery in the clinical management of SBS.

